# Male attractiveness in fruit flies is influenced by previous exposure of females to males of different attractiveness

**DOI:** 10.1038/s41598-024-66930-0

**Published:** 2024-07-16

**Authors:** Laure-Anne Poissonnier, Etienne Danchin, Guillaume Isabel

**Affiliations:** 1 Centre de Recherches Sur La Cognition Animale (CRCA), Centre de Biologie Intégrative (CBI), UMR 5169, CNRS, Université de Toulouse Midi-Pyrénées, Toulouse, France; 2 Laboratoire Evolution et Diversité Biologique, University of Toulouse 3, Toulouse, France

**Keywords:** Behavioural ecology, Sexual behaviour

## Abstract

Mate choice is a crucial decision in any animal. In terms of fitness, the best mate is the one that leads to the most abundant and productive offspring. Pairing with a low-quality mate would reduce fitness, generating selection for accurate and subtle mate choice in all animal species. Hence, mate choice is expected to be highly context dependent, and should depend on other potential options. For instance, a medium-quality male can constitute the best option when all other males are in poorer condition, but not when there are better-quality males available. Therefore, animals are predicted to gather information about their social context and adapt their mate choice to it. Here, we report on experiments in which we manipulated the social environment of females of *Drosophila melanogaster* and found that after encountering a high or a low-quality male, they take more or less time to accept copulation with another male, suggesting that females adapt their mating strategy to their social context. We also report on a similar effect in *D. biarmiceps*. Thus, male attractiveness appears to depend on the quality of recently met males, suggesting that male attractiveness is subjective, indicating plastic and context dependent mate choice.

## Introduction

It has long been assumed that humans are rational decision-makers who, when faced with a choice between different options, will usually choose the best option^1^. It is tempting to think that we are good at evaluating options independently from each other, and that each option has a fixed, stable value. In reality, however, the perceived value of each option largely depends on the context and prior experience^2^. In particular, our decisions are more often than not influenced by cognitive biases, *i.e.* the altered processing of information as the result of our background emotional state or prior experience^1^. A notable illustration of this is the decoy effect. The decoy effect is a cognitive bias where the presence of an inferior choice modifies the behavior of people towards other, superior options in a multiple-choice situation. The evidence of the decoy effect has been seen in a wide variety of context, and has been studied in real-world elections as well as a high number of behavioral economics scenarios, including evolutionary game theory^3^.

Similarly, animal decision-making is known to be influenced by cognitive biases^4,5^. In particular, the perceived value of various options depends on previous experience^6,7^. Subtle context-dependent decision-making has been described in most vertebrate sub-taxa (blue and grey jays^8^, humming birds^9^, frogs^10^, fish^11^, dolphins^12^) as well as in many invertebrates (*e.g.* honeybees^13^, bumblebees^14^, ants^15^). For instance, stressed honeybees show pessimistic biases when having to classify an ambiguous odour stimulus^16^. In a similar task using visual cues, Bumblebees *Bombus terrestris* that received an unexpected food reward before being tested later display consistent optimistic biases^1^. Similarly, stressed (*i.e.* shaken) fruit flies *D. melanogaster* show a pessimistic bias in an ambiguous odour binary choice task, while unstressed flies did not ^17^.

One of the most crucial decision in a sexually reproducing organism is the choice of a sexual partner, and its impact on fitness has been analysed extensively^18^. In that context, if animals decided whom to mate with based on a fixed value assigned to a mate, which could depend on criteria such as size, plumage colour, or courtship quality, then the presence of other mates should not affect their decision. Lea et al. in 2019 showed that this wasn’t the case for Tungara frogs^10^, because adding a third male option modified the preference between two other males.

In this article, we investigated similar questions in Drosophila, a model that would permit the exploration of underlying neuronal mechanisms of decision-making and subjectivity. The evidence of the impact of the social environment in Drosophila’s behaviour is accumulating. An example of this is the modification of female mating preference between 2 male strains according to the composition of the male group in both strains^19^. It has also been shown that females’ choice can be impacted by the choice of other females: they prefer to mate with males that have phenotypes similar to the ones that they saw being selected by other females^20,21^.

In this study, we checked whether the knowledge of the existence of males of different quality was sufficient to affect the speed at which females accepted to mate. Despite the evolutionary importance of mate choice^4^, its underlying cognitive mechanisms remain largely unknown in most species. A better understanding of those mechanisms is however crucial to understand mate selection and gene transmission. Here, we tested whether the mating behavior of Drosophila females is affected by alternative male options, exploring whether a component of the social context could affect male attractiveness, without learning from other females’ choice or direct interactions with the males. We tested the effects of 2 types of cognitive biases: the presence of different options at the moment of the choice, as well as previous experience, which can modify the perceived value of future options^2,5^. Importantly, we used virgin females separated from males from emergence, to prevent the existence of other cognitive biases from previous experience. We expect females to be biased by their knowledge of other options, and the males to be evaluated in comparison to other males, and not purely on objective criteria. We expect this bias to be reflected in the time taken to accept copulation.

## Methods

### Breeding and fly collection

Flies were raised in 30 mL vials containing 8 mL corn flour-agar-yeast medium at 25 °C and 60% humidity, on a 12/12 day/night cycle. Flies of the wild-type Canton S line (high attractiveness HA) were provided by the Thomas Preat’s lab, and yellow lines w + y (low attractiveness LA), and ebony line *TM2/TM6b* (intermediate attractiveness Int-A) by Drosophila Bloomington stock center. They were maintained at 25 °C and 60% humidity, on a 12/12 day/night cycle in vials in which we put 6 mature males and females for 48–72 h. All demonstrator or subject flies were collected within ≤ 90 min after emergence and kept in single-sex vials to ascertain virginity until experiment at the age of 3–4 days. Females were kept in groups of 7 and males in groups of 14, as in Danchin et al.^20^.

### Experimental procedure

Experiments unfolded in a circular arena made of transparent resin of 1.1 cm radius and 4 mm in height, divided in two equal compartments by a transparent sliding door through which the focal CS female could see (but not interact with) the male. Flies were introduced in the arena and removed through gentle aspiration via a mouth aspirator.

*Experiment 1:* The first step was to select males of varying attractiveness. We ranked male attractiveness by giving a wild type Canton S (CS) virgin female the choice between 2 males of three contrasted phenotypes (selected from pilot experiments) in a small circular arena and recording with which male she mated. N = 60 females.

*Experiment 2*: We then used that ranking to investigate the effects of exposure to another male during, or 10 or 30 min prior to a mating test. The delayed 10 and 30 min conditions were used to investigate whether exposure could still have an effect after the males were removed (a sort of memory effect). Specifically, we exposed CS females to a highly attractive (HA) or an unattractive (LA) male for 10 min and assessed the attractiveness of an intermediate attractive (Int-A) male by measuring the latency between the beginning of male courtship and the onset of copulation that followed. To assess male attractiveness, we filmed the experiment for 30 min after introducing the Int-A (Ebony) male. We measured the mating latency, recorded from the beginning of courtship (when the male first wing flaps to court the female) to the onset of copulation that followed. We explored 4 conditions. In condition a), the exposure to a LA or a HA male unfolded while the virgin CS female was choosing to copulate with an Int-A male (without any prior experience, Fig. 2a). In condition b) and c) the introduction of the Int-A male occurred 10 min or 30 minutes after the exposure to a LA or HA male, so that no other male than the Int-A male was visible during the test (Fib2b,c). d) A final condition played the role of a control in that it replicated condition b) but with an opaque paper preventing the female from seeing the male during exposure. N = 25 females for each sub-treatment.

*Experiment 3*: This experiment tests whether the cognitive biases measured in Experiment 2 could affect the decision-making process in a choice between 2 males in binary tests. Lea and Ryan (2015) found that the presence of a third, suboptimal male affects mate choice between 2 males in Tungara frogs^10^, revealing a decoy effect leading to deviation from rational choices, as is found in humans^2,7,22^. To investigate the existence of similar phenomena in Drosophila, we recorded the proportion of CS females mating with HA or Int-A males in a binary choice situation, 10 min after observing either no male, a HA, or a LA male. N = 60 females.

*Experiment 4*: The phenotypes we used in the previous experiments were artificial laboratory phenotypes created in Drosophila. We wanted to verify whether the cognitive biases documented in experiments 1 to 3 persisted when using more natural variation in attractiveness, and find a phenotype linked to variation in attractiveness that could be used in other Drosophila species. Damaged wings are common in nature. Females likely associate such damages with low fitness^23^ as it impairs flight and courtship song, as these are produced through wing-flapping. We thus mimicked ‘damaged’ wings by gently shaking males in wet and sticky food media, resulting in their wings being stuck and folded. In the wing condition experiments, as a few males were able to unstuck their wings after a while in less than 10 min, we adopted a 5 min demonstration period to ensure that all males kept stuck wings during the whole demonstration. We previously checked that a 5 min observation period was sufficient to induce a cognitive bias in the LA *vs* HA conditions (coxph, *P* = 0.03). We repeated experiment 2, using exposure to such CS males with stuck wings in comparison with exposure to normal CS males or no male, the last condition providing a control for a possible effect of familiarity (Fig. 4a). N = 25 females. Furthermore, as this protocol is applicable even in the absence of known phenotype affecting genetic mutants, we applied it to two other Drosophila species, wild caught *Drosophila simulans* and *Drosophila biarmiceps* reared for at least a year in the same conditions as *D.melanogaster*, using the wing conditions protocol.

### Data analysis

Sample sizes in the first and third experiment were of 60 females tested per condition, and 25 per condition in all other experiments. Final sample sizes may however be a bit lower due to rare instances in which no copulation occurred during the 30 min of the mate choice test (number of females that did not mate in 30 min was 5 out of 190 in experiment 2, and 52 out of 225 in experiment 4). All statistics were done with RStudio 2022.02.2^24^. Survival analysis were done with the ‘survival’ package^3^, as " Survival analysis concerns the follow-up in time of individuals from an initial experience or exposure until a discrete event”^4^, here copulation. The binomial test of experiment 3 was done with the binom.test function, comparing the proportion of mating in the LA males exposed to the proportion of the HA males exposed, with alternative = two sided and conf.level = 0.95.

## Results and discussion

### Experiment 1: sexual attractiveness of various male phenotypes

**Figure 1:**
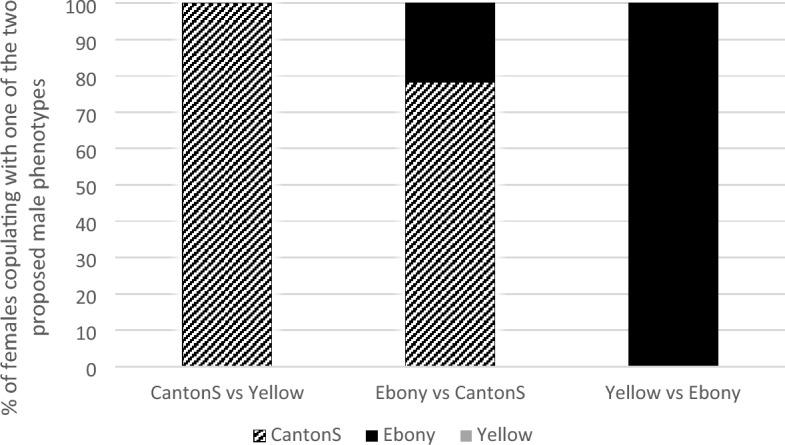
Ranking female preference for three types of males in binary choices. Canton S Drosophila females prefer CS males (*i.e.* wild type male) over Yellow, and Ebony over Yellow males. Percentage of males of each phenotype selected by a CS female in a choice situation between two males. Sample sizes are 60 in all three cases. This provides the following rank in attractiveness from low to high: Yellow = low attractiveness males (LA) < Ebony: intermediate attractiveness male (IntA) < Canton S: highly attractive males (HA).

Wild type Canton S (CS) Drosophila females preferentially mated with CS males, never mated with males with a yellow body color (*i.e.* carrying the *yellow* allele, thereafter called “yellow”), and preferred CS over *TM2/TM6b* ebony-colored males (*i.e.* carrying the *ebony* allele via *TM2/TM6b* balancers line, thereafter called “Ebony”; Fig. 1). Male attractiveness thus decreased from CS (high attractiveness), to Ebony (intermediate attractiveness), to Yellow (low attractiveness). This ranking is consistent with the fact that males carrying the yellow genes have a lower mating success^25^. Those results could result from male-male competition rather than female presence, wild type Canton S being potentially more competitive than yellow males. However, results from experiments 2 and 4, where no male competition is present, combined with experiment 3 prove that, while the role of male competition is not excluded, female preference plays a crucial role in mating choice.

### Experiment 2: male attractiveness is relative to the quality of other encountered males

**Figure 2:**
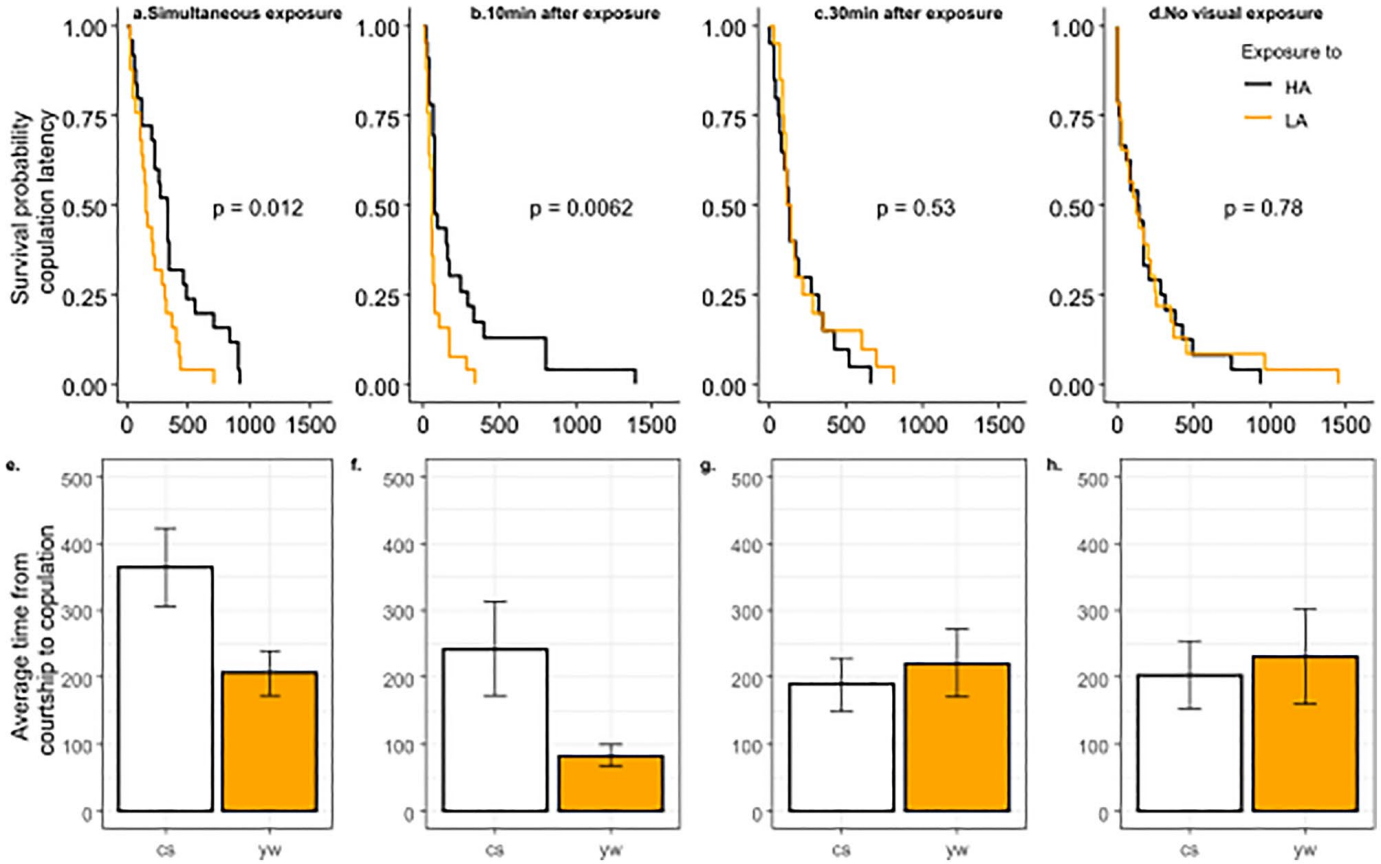
Survival probabilities of (top row **a**, **b**, **c**, **d**) and mean latencies (bottom row **e**, **f**, **g**, **h**) in copulation by CS females as a function of their exposure to males of different qualities. Times in seconds. Exposures consisted of a Yellow or a Canton S male present in the other half of the experimental arena separated by a transparent partition. (**a**) Survival curves of the latency of a CS female to mate with an Ebony (IntA) male in the simultaneous presence of another male. (**b**) and (**c**) survival curves of the latency of a CS female to mate with an IntA male, 10 (**b**) or 30 (**c**) minutes after a 10 min exposure to a HA (Canton S in black) or LA (Yellow, in orange) male. (**d**) Control replicating condition (**b**), but with an opaque partition preventing the female from seeing the demonstration male (acoustic and/or olfactory cues were however not excluded). N = 20–25 females per condition.

If attractiveness stems from a fixed value based on criteria such as size, colour, or courtship quality, the exposure should not affect mating decisions. We found that previous or simultaneous exposure to males of different qualities affected the speed at which females accepted the intermediate attractiveness (IntA) males (Fig. 2a,b,e,f). Interestingly, virgin females that had never been in contact with males prior to the experiment mated quicker with the IntA males in the presence of a LA than a HA male visible through the transparent door during the test (Fig. 2a,e, coxph, *P* = 0.012). Therefore, the attractiveness of the IntA male with which the female copulates appeared to be dependent on the attractiveness of the male observable through the transparent door during the test. IntA males were more attractive in the presence of a less attractive male, than in the presence of a more attractive HA male, suggesting that the attractiveness is *subjective* to the social context. This results is reminiscent of decoy effects, cognitive biases emerging from what is perceived during decision-making, impacting decision-making processes^7,22^. It remains to be established whether it is the presence of the LA or the HA male that is affecting the female behavior, or both. Next, we tested whether previous experience affects the choice while the other options were not present anymore (memory effect). We found that females mated quicker after seeing a LA than a HA male when tested 10 min after exposure (Fig. 2b,f, coxph, *P*-value = 0,0062), but not after 30 min (Fig. 2c,g, coxph, *P*-value = 0,53), suggesting that this effect is relatively short lived, but exists nonetheless in the absence of the male. Finally, the control condition d) supports the fact that females are using visual cues to assess male quality, as the bias was not present anymore if an opaque separation was added to prevent observation (Fig. 2d,h, coxph, p-value = 0,78). This suggests that female mating decisions can be impacted by the visual cues of a single male in Drosophila, while previous work investigated group composition effects and modulation of sex pheromone^19,26^.

These results are consistent with recent findings showing that cognitive biases also affect insects such as honey bees^16^ and bumblebees *B. terrestris* biases^27^.Stressed (*i.e.* shaken) fruit flies *D. melanogaster* showed a pessimistic bias in an ambiguous odour binary choice task, while unstressed flies did not^17^. In the same vein, in ants, the perception of food quality is subjective to their previous experience and expectations^15^. Our results thus add that this subjective perception can also bias a judgment in the area of sex, raising the question of whether this bias has an impact on mate choice, and consequently on evolution?

### Experiment 3: previous exposure to different male phenotypes impacts actual female binary choice

**Figure 3:**
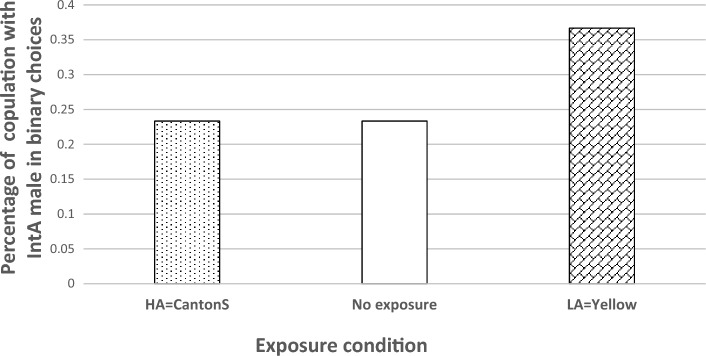
Percentage of copulations with Inta-A males (as opposed to HA males) in binary choices by CS females, 10 min after a 10 min exposure to a HA male, no male, or a LA male (from left to right). Sample sizes for each treatment was 60 females. In the HA and no exposure conditions, the same number of females copulated with the IntA male (14 out of 60). However, after exposure to a LA male, the number of females that selected IntA males was significantly higher (binomial test, p-value = 0.02).

In all test, as in Experiment 1, we found a preference for the HA males (Fig. 3). However, such female preference for HA males was reduced by about 15% when females saw a LA male compared to a HA male (Fig. 3). Such a cognitive bias would have the potential to affect sexual selection significantly. Seeing a LA male reduced the perceived difference between the IntA and HA males significantly (binomial test, *P*-value = 0,02), as was found in Tungara frogs^10^. This phenomenon might thus be more widespread in the animal kingdom than usually thought, begging for more investigation on this topic.

### Experiment 4: a more ecologically relevant phenotype, damaged wings, also leads to the same cognitive *bias*

**Figure 4:**
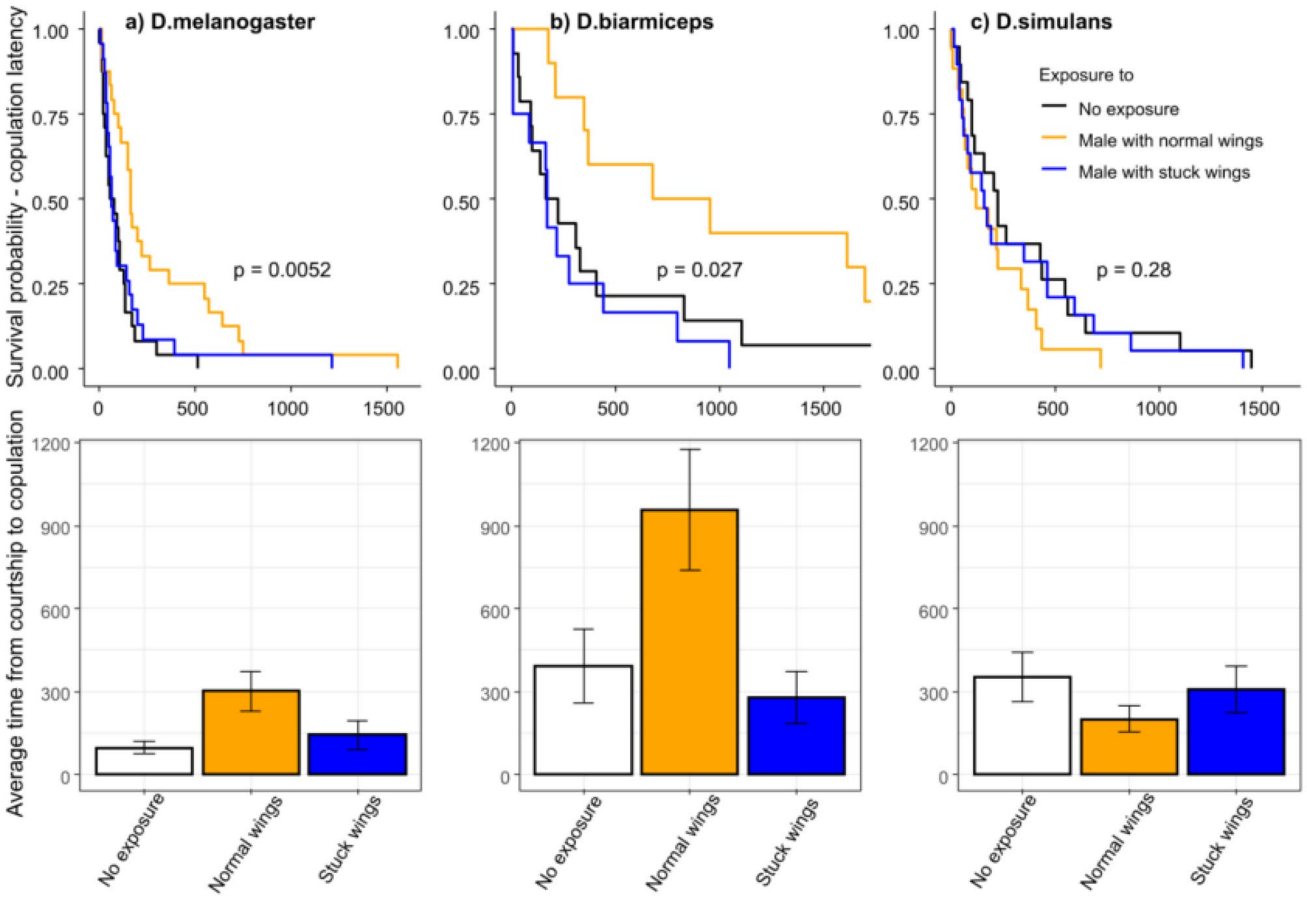
Latency survival curves (top row) and mean (bottom row) of females to mate with a male (with normal wings), 10 min after a 5 min exposure to either no male (black line and white bars), a male with normal wings (in orange), or a male with stuck wings (in blue). Times in seconds. (**a**) in *Drosophila melanogaster*, (**b**) in *Drosophila biarmiceps*, (**c**) in *Drosophila simulans*. N = 25 females per condition.

We found that if females did not see any male, or were exposed to a CS male with stuck wings, they mated quickly, while if they saw a HA male with normal wings during exposure and test they took longer (Fig. 4a, coxph, *P* = 0.0052). This could be interpreted as if the female has no other option or a worse option, the IntA option appears good, or that comparing two similar HA males could take longer than comparing nothing *vs* something, or a medium *vs* a bad option.

Furthermore, as this protocol is applicable even in the absence of known phenotype affecting genetic mutants, we applied it to two other Drosophila species, using the wing conditions protocol. We found the same bias in *D. biarmiceps*, but not *D. simulans* (Fig. 4b,c, Fig. 2a,e, coxph, *P* = 0.027 and 0.28, respectively). This may, for instance, result from differences in their ecology, as in *D. simulans* remating might be common and beneficial, while it is costly in other species^28^. Hence, mate choice may be less constrained and crucial in *D. simulans* females, compared to species in which females likely copulate with one/a few male in their lifetime which could explain why we did not find the cognitive bias in *D. simulans*.

In conclusion, it appears that male attractiveness is subjective and depends on the social context in Drosophila, which has a significant impact on sexual evolution. Together with the fact that *Drosophila melanogaster* performs mate copying^20,21^, and that the group composition in different genotypes affects mating behaviour^19,26^, this result reinforces the idea that Drosophila species are highly sensitive to their social context and do collect many forms of social information, at least in the context of mate choice. Because we cannot ask animals feedbacks on how they feel, behavioral protocols such as the one we develop here can allow us to quantify the subjective value attributed to an object/situation providing us with a way to study subjectivity in animal mate choice. Applying this protocol, as we do here, to the *D. melanogaster* model with its suite of powerful genetic tools may open an avenue of research to study the underlying neural mechanisms of subjectivity and animal mating behavior.

## Data Availability

The raw data is available on Dryad DOI https://datadryad.org/stash/share/qbiyIVfJjXCc69HWShBlavOJ5coM42URQNFX-W1Ebgo
