# Author Correction: Male attractiveness in fruit flies is influenced by previous exposure of females to males of different attractiveness

**DOI:** 10.1038/s41598-025-95428-6

**Published:** 2025-03-27

**Authors:** Laure-Anne Poissonnier, Etienne Danchin, Guillaume Isabel

**Affiliations:** 1 Centre de Recherches Sur La Cognition Animale (CRCA), Centre de Biologie Intégrative (CBI), UMR 5169, CNRS, Université de Toulouse Midi-Pyrénées, Toulouse, France; 2 Laboratoire Evolution et Diversité Biologique, University of Toulouse 3, Toulouse, France

Correction to: *Scientific Reports* 10.1038/s41598-024-66930-0, published online 16 July 2024

The original version of this Article contained errors in the title of the paper. As a result,

“Male attractiveness is subjective to exposure to males of different attractiveness in fruit flies.”

now reads:

“Male attractiveness in fruit flies is influenced by previous exposure of females to males of different attractiveness.”

The original Article has been corrected.

